# Structural Correlates of Reading the Mind in the Eyes in Autism Spectrum Disorder

**DOI:** 10.3389/fnhum.2017.00361

**Published:** 2017-07-12

**Authors:** Wataru Sato, Shota Uono, Takanori Kochiyama, Sayaka Yoshimura, Reiko Sawada, Yasutaka Kubota, Morimitsu Sakihama, Motomi Toichi

**Affiliations:** ^1^Department of Neurodevelopmental Psychiatry, Habilitation and Rehabilitation, Kyoto University Kyoto, Japan; ^2^Brain Activity Imaging Center, Advanced Telecommunications Research Institute International Kyoto, Japan; ^3^Health and Medical Services Center, Shiga University Hikone, Japan; ^4^Rakuwa-kai Otowa Hospital Kyoto, Japan; ^5^Faculty of Human Health Science, Kyoto University Kyoto, Japan; ^6^The Organization for Promoting Neurodevelopmental Disorder Research Kyoto, Japan

**Keywords:** autism spectrum disorder (ASD), reading the mind in the eyes test, structural magnetic resonance imaging (MRI), temporoparietal junction (TPJ), voxel-based morphometry (VBM)

## Abstract

Behavioral studies have shown that individuals with autism spectrum disorder (ASD) have impaired ability to read the mind in the eyes. Although this impairment is central to their social malfunctioning, its structural neural correlates remain unclear. To investigate this issue, we assessed Reading the Mind in the Eyes Test, revised version (Eyes Test) and acquired structural magnetic resonance images in adults with high-functioning ASD (*n* = 19) and age-, sex- and intelligence quotient-matched typically developing (TD) controls (*n* = 19). On the behavioral level, the Eyes Test scores were lower in the ASD group than in the control group. On the neural level, an interaction between group and Eyes Test score was found in the left temporoparietal junction (TPJ). A positive association between the Eyes Test score and gray matter volume of this region was evident in the control group, but not in the ASD group. This finding suggests that the failure to develop appropriate structural neural representations in the TPJ may underlie the impaired ability of individuals with ASD to read the mind in the eyes. These behavioral and neural findings provide support for the theories that impairments in processing eyes and the ability to infer others’ mental states are the core symptoms of ASD, and that atypical features in the social brain network underlie such impairments.

## Introduction

Autism spectrum disorder (ASD) is a behaviorally-defined neurodevelopmental disorder primarily characterized by impaired social interactions (American Psychiatric Association, 2013). One of the most evident features of social impairment is a deficit in eye processing (Baron-Cohen, 1995). Several behavioral studies have found that individuals with ASD, compared with typically developing (TD) individuals, are less likely to use eye direction as a cue that others are thinking (Baron-Cohen and Cross, 1992) and to infer another person’s desires and goals (Baron-Cohen et al., 1995).

Researchers have developed a test to measure the ability to read others’ minds in their eyes, the Reading the Mind in the Eyes Test, revised version (hereafter, Eyes Test; Baron-Cohen et al., 2001) to measure and quantify this impairment in individuals with ASD. In this test, participants are presented with a photograph depicting only the eye region of a person and asked to choose one of four adjectives or phrases to describe the mental state of the person. Using the Eyes Test, several studies have consistently reported worse performance in adults, adolescents and children with ASD than in TD individuals (Baron-Cohen et al., 1997; Golan and Baron-Cohen, 2006; Lombardo et al., 2007; Losh et al., 2009; Sachse et al., 2014; Vogindroukas et al., 2014). A previous study with TD participants reported high reliability for the Eyes Test, even over a 1-year period (Fernández-Abascal et al., 2013). Behavioral genetics studies revealed a genetic influence on Eyes Test performance (Rodrigues et al., 2009; Warrier et al., 2013; Gong et al., 2014). Taken together, these behavioral data suggest that individuals with ASD have a stable, possibly genetically determined impaired ability to read the mind in the eyes, as measured using the Eyes Test.

Several previous functional neuroimaging studies using magnetic resonance imaging (MRI) have investigated neural activity to understand the neural mechanisms underlying the impaired ability to read the mind in the eyes in adult and adolescent individuals with ASD (Baron-Cohen et al., 1999; Holt et al., 2014) and in adult and adolescent individuals with the genetic characteristics of ASD (i.e., parents and siblings of individuals with ASD; Baron-Cohen et al., 2006; Holt et al., 2014) while performing the Eyes Test (Baron-Cohen et al., 1999) or the Eyes Test vs. sex judgments (Baron-Cohen et al., 2006; Holt et al., 2014). These studies found that the ASD group shows reduced activation in some brain regions that are clearly activated in TD individuals, such as the temporoparietal junction (TPJ, the boundary between the temporal and parietal lobes, including the posterior middle and superior temporal gyri and the inferior parietal lobule; Baron-Cohen et al., 2006; Geng and Vossel, 2013; Holt et al., 2014), amygdala (Baron-Cohen et al., 1999), and inferior frontal gyrus (IFG; Baron-Cohen et al., 1999; Holt et al., 2014). To complement these findings, several functional neuroimaging studies tested only TD adult individuals during performance of the Eyes Test or the Eyes Test vs. sex judgments (Russell et al., 2000; Platek et al., 2004; Adams et al., 2010; Castelli et al., 2010; Mascaro et al., 2013; for a review see Schurz et al., 2014). These studies reported rather consistent activation in some of these brain areas, including the TPJ (Russell et al., 2000; Platek et al., 2004; Adams et al., 2010; Castelli et al., 2010; Mascaro et al., 2013), amygdala (Castelli et al., 2010; Mascaro et al., 2013), IFG (Russell et al., 2000; Adams et al., 2010; Castelli et al., 2010; Mascaro et al., 2013) and dorsomedial prefrontal cortex (dmPFC; Platek et al., 2004; Adams et al., 2010; Castelli et al., 2010; Mascaro et al., 2013). These data suggest that reduced activation in these brain regions may be associated with decreased ability in individuals with ASD to infer the mental states of others by viewing the eye region.

However, the structural neural correlates of stable impairment on the Eyes Test in individuals with ASD remain unknown. To date, no structural MRI study has investigated this issue in individuals with ASD. A recent structural MRI study reported that Eyes Test scores were positively associated with gray matter volume in some brain regions, including the TPJ and dmPFC, in TD individuals (Sato et al., 2016). These data suggest that, in TD, the structural neural substrates of Eyes Test performance are located within distinct brain regions, and that abnormalities in these regions might be detectable in individuals with ASD. Several previous structural MRI studies have shown that gray matter volume was reduced in some brain regions, including the TPJ (Hadjikhani et al., 2006; Craig et al., 2007; Scheel et al., 2011; Ecker et al., 2012; Greimel et al., 2013; Mueller et al., 2013; David et al., 2014) and dmPFC (Abell et al., 1999; Hadjikhani et al., 2006), in individuals with ASD relative to TD controls, although the results were not consistent across studies (for a review see Yang et al., 2016). Furthermore, previous structural MRI studies found abnormal relationships between social behaviors and brain structures in individuals with ASD, such as weaker and negative correlations between the processing of social stimuli and gray matter volume in the fusiform gyrus (Dziobek et al., 2010; Trontel et al., 2013) and amygdala (Dziobek et al., 2006), although the patterns were inconsistent (David et al., 2014). A recent study found that gray matter volume and functional activation was decreased in brain regions, including the dmPFC, in individuals with ASD relative to TD controls (Carlisi et al., 2017). Based on these findings, we hypothesized that the association between the Eyes Test score and gray matter volume in areas generally activated in TD individuals would be weaker in individuals with ASD relative to TD controls.

To test this hypothesis, we acquired MRI data from and administered the Eyes Test to high-functioning adults with ASD, who had no comorbidities and were not taking medication. We recruited age-, sex-, intelligence quotient (IQ)-matched TD controls. We analyzed the group differences in the association between the Eyes Test score and gray matter volume using voxel-based morphometry (VBM).

## Materials and Methods

### Participants

The ASD group consisted of 19 adults with ASD (5 females, 14 males; mean ± *SD* [range] age = 28.1 ± 9.0 [19–53] years). All were native Japanese. Diagnoses were made by two psychiatrists with expertise in developmental disorders (MT and SY) using DSM-IV-TR (American Psychiatric Association, 2000) criteria. The diagnoses were accepted only if they completed an agreement. Neurological and psychiatric problems other than those associated with ASD were ruled out. None of the participants was taking medications. Full-scale IQs, measured by the Wechsler Adult Intelligence Scale, third edition (WAIS-III; Nihon Bunka Kagakusha, Tokyo, Japan) fell within the normal range in all participants in the ASD group (mean ± *SD* [range] = 112.3 ± 13.7 [86–134]). Symptom severity was assessed quantitatively using the Childhood Autism Rating Scale (Schopler et al., 1986) in some participants (*n* = 13), and their scores (mean ± *SD* [range], 25.0 ± 3.1 [18.0–30.5]) were comparable to those of previous studies in high-functioning individuals with ASD (Koyama et al., 2007; Uono et al., 2011; Sato et al., 2012).

The TD group consisted of 19 native Japanese adults who were carefully matched in terms of age (mean ± *SD* [range] = 23.3 ± 3.8 [19–32] years; *t*_(36)_ = 1.52, *p* > 0.1), sex (5 females, 14 males; *χ*^2^_(1)_ = 0.00, *p* > 0.1), and full-scale IQ (mean ± *SD* [range] = 114.8 ± 7.0 [101–124]; *t*_(36)_ = 0.60, *p* > 0.1) with the ASD group. A psychiatrist or psychologist administered a short structured diagnostic interview using the Mini-International Neuropsychiatric Interview (Sheehan et al., 1998); no neuropsychiatric problem was detected in any participant. The TD group included participants used in a previously published report (Sato et al., 2016).

All participants were right-handed, as assessed by the Edinburgh Handedness Inventory (Oldfield, 1971) and had normal or corrected-to-normal visual acuity. After the procedures were fully explained, all participants provided written informed consent for participation. This study was approved by the local ethics committee of the Primate Research Institute, Kyoto University, and conducted in accordance with the approved guidelines.

### Task

**Figure 1:**
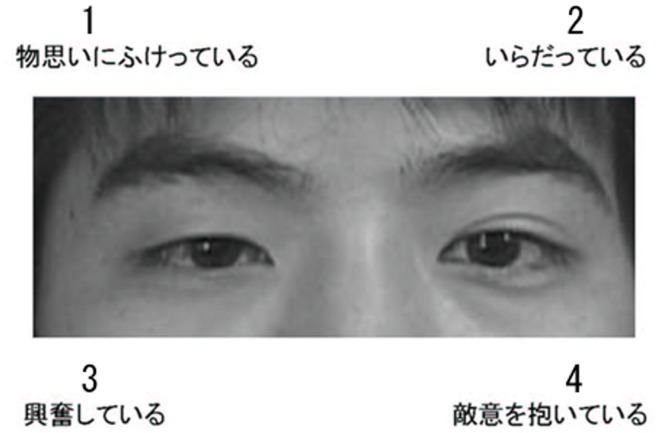
An illustration of the Asian version of the Eyes Test stimuli. The choice options were: (1) *monoomoinifuketteiru* (pensive); (2) *iradatteiru* (irritated); (3) *koufunshiteiru* (excited); and (4) *tekiiwoidaiteiru* (hostile). The correct answer (i.e., that selected most frequently in the validation task) was option 1.

Because our participants were all Japanese, the Asian version of the Eyes Test (Adams et al., 2010) was used. An illustration of stimuli is shown in Figure 1; the model provided written consent for the presentation of his photograph. The Asian version of the test assessed the same mental states as the original version (Baron-Cohen et al., 2001). As in the original version, the Asian test consisted of 36 photographs depicting only the eye region; however, the individuals depicted were East Asian rather than Caucasian. The photographs were collected from divergent sources, including magazines and databases of amateur models. The Asian version retained the four mental state terms (e.g., irritated; one target and three foils) accompanying each photograph used in the original version. The terms were translated into Japanese and the validity of the translation was confirmed through back translation. A previous study tested the Asian version on 61 Japanese participants and found an accuracy of more than 73% (Adams et al., 2010). Functoinal MRI assessment of the Asian version in Japanese participants found that, as in the original version, the test activated brain regions related to mind reading, including the TPJ, IFG and dmPFC (Adams et al., 2010). Although the reliability of the Asian version has not been tested, several studies using the original version have reported that reliability was high (Fernández-Abascal et al., 2013; Vellante et al., 2013; Prevost et al., 2014; Khorashad et al., 2015).

The task was controlled by SuperLab Pro 2.0 (Cedrus, San Pedro, CA, USA), implemented on a Windows computer (HP Z200 SFF; Hewlett-Packard, Tokyo, Japan). The stimuli were presented on a 19-inch CRT monitor (HM903D-A; Iiyama, Tokyo, Japan). The photographs subtended visual angles of 12.0° horizontally × 4.8° vertically.

### MRI Acquisition

Image scanning was performed on a 3-T MRI system (MAGNETOM Trio, A Tim System, Siemens, Erlangen, Germany) at the ATR Brain Activity Imaging Center using a 12-channel head coil. A forehead pad was used to stabilize the head position. A T1-weighted high-resolution anatomical image was obtained using a magnetization-prepared rapid-acquisition gradient-echo sequence (repetition time = 2250 ms; echo time = 3.06 ms; inversion time = 1000; flip angle = 9°; field of view = 256 × 256 mm; voxel size = 1 × 1 × 1 mm).

### Behavioral Data Analysis

Behavioral data were analyzed using SPSS 16.0J (SPSS Japan, Tokyo, Japan). Eyes Test scores were analyzed using a *t*-test between groups and analysis of covariance (ANCOVA) with group (TD and ASD) as an effect-of-interest factor and age, sex and full-scale IQ as effect-of-no-interest covariates. A *p-value < 0.05 was* considered significant.

### Image Analysis

Image analysis was performed using the statistical parametric mapping package, SPM8^1^ and the VBM8 toolbox^2^ implemented in MATLAB R2012b (MathWorks Inc., Natick, MA, USA). First, the images were preprocessed using the VBM8 toolbox using default settings. All structural T1 images were segmented into gray matter, white matter and cerebrospinal fluid using an adaptive maximum a posteriori (AMAP) approach (Rajapakse et al., 1997). Intensity of homogeneity on the image was modeled as slowly varying spatial functions and thus corrected in the AMAP estimate. The segmented images were then used for a partial volume estimate using a simple model with mixed tissue types to improve segmentation (Tohka et al., 2004). Furthermore, a spatially adaptive non-local means denoising filter was applied to deal with spatially varying noise levels (Manjón et al., 2010). A Markov Random Field cleanup was used to improve image quality. The gray matter images in native space were subsequently normalized to the standard stereotactic space defined by the Montreal Neurological Institute using the diffeomorphic anatomical registration using the exponentiated lie algebra algorithm approach (Ashburner, 2007). We used predefined templates provided in the VBM8 toolbox that were derived from 550 healthy brains in the IXI-database^3^. The resulting normalized gray matter images were modulated using Jacobian determinants with non-linear warping only (i.e., m0 image in the VBM8 outputs) to exclude the effect of total intracranial volume. Finally, the normalized modulated gray matter images were resampled to a resolution of 1.5 × 1.5 × 1.5 mm and smoothed using a 12-mm full-width at half-maximum (FWHM) isotropic Gaussian kernel based on the recommendation of the VBM method, where FWHM is typically between 4 mm and 12 mm (Ashburner, 2010). We selected the relatively large smoothing kernel because it improved the normality of the distribution of the data and increased the validity of the parametric statistics for our relatively small sample size (Ashburner and Friston, 2000).

To identify the brain regions associated with between-group differences in the association between the Eyes Test score and gray matter volume, we performed a general linear model analysis with group (TD and ASD) and group-interacted Eyes Test score (Eyes Test score for each TD and ASD group; the Eyes Test scores were overall mean centered) as the effect-of-interest factors, and age, sex and full-scale IQ as the effect-of-no-interest covariates (Supplementary Figure S1). Such modeling is similar to that exploring the interactions between categorical and continuous variables in analyses of neuroimaging data (Poldrack et al., 2011) and is mathematically equivalent to the conventional model with group and Eyes Test score as the main effects, with interactions and covariates. Our prediction was related to the interaction between group and Eyes Test score (i.e., TD Eyes Test score vs. ASD Eyes Test score). Simple effect analyses were conducted to follow up significant interactions. The effects were tested using *T*-statistics. Voxels were deemed significant if they reached the extent threshold of *p* < 0.05, with family-wise error correction for multiple comparisons over the search volume, with a cluster-forming threshold of *p* < 0.001 (uncorrected). We selected the regions previously reported across multiple studies to show activation in the TD individuals as they completed the Eyes Test as regions of interest (ROIs). The ROIs specifically included the TPJ, amygdala, IFG and dmPFC. We performed a small-volume correction (Worsley et al., 1996) for these ROIs, and search volume was restricted by constructing anatomical masks of a 12-mm-radius sphere centered on coordinates taken from previous studies. Information on coordinates was derived from Adams et al. (2010), who reported significant activation during the Asian version of the Eyes Test in the bilateral TPJ (*x*-48, *y*-48, *z*16; *x*52, *y*-48, *z*14), bilateral IFG (*x*-54, *y*32, *z*-4; *x*58, *y*30, *z*6) and left dmPFC (*x*-4, *y*16, *z*56). Information on the bilateral amygdala (*x*-26, *y*-11, *z*-7; *x*20, *y*-8, *z*-7) that was not reported by Adams et al. (2010) was derived from Baron-Cohen et al. (1999). Note that these ROIs were not based on our dataset results, rather they were selected based on the activation evident in previous fMRI studies. We thus sought to avoid circular or non-independent analyses (see Kriegeskorte et al., 2009). Other areas were corrected for the entire brain volume (*k* > 850). The brain structures were labeled anatomically and identified according to Brodmann’s areas (BAs) using the automated anatomical labeling atlas (Tzourio-Mazoyer et al., 2002) and Brodmann maps^4^, respectively, using MRIcron software^5^. The relationship between gray matter volume and the Eyes Test for each group was illustrated by plotting the gray matter values extracted at peak voxels against test scores after adjusting for the effects-of-no-interest by regressing out age-, sex- and full-scale IQ-related variance.

## Results

### Eyes Test Scores

**Figure 2:**
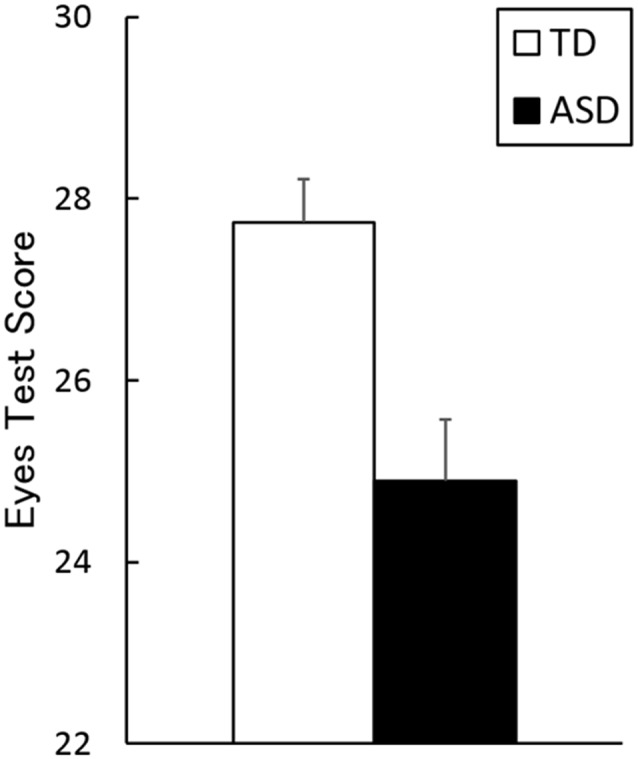
Mean (with *SE*) scores on the Eyes Test in the typically developing (TD) and autism spectrum disorder (ASD) groups.

The mean ± *SE* (range) Eyes Test scores for TD and ASD groups were 27.3 ± 0.5 (23–30) and 24.9 ± 0.7 (18–29), respectively (Figure 2). The *t*-test revealed a significant group difference, indicating higher performance by the TD group than by the ASD group (*t*_(36)_ = 3.43, *p* < 0.005, *r* = 0.44). The ANCOVA with group as a factor and age, sex and full-scale IQ as covariates confirmed the main effect of group (TD > ASD; *F*_(1, 33)_ = 12.11, *p* < 0.005, $\eta_{\text{p}}^{2}$ = 0.22). The covariate effect of full-scale IQ was significant (*F*_(1, 33)_ = 4.70, *p* < 0.05, $\eta_{\text{p}}^{2}$ = 0.13), indicating a positive association between the Eyes Test score and full-scale IQ, and the covariate effect of sex reached marginal significance (*F*_(1, 33)_ = 4.00, *p* < 0.1, $\eta_{\text{p}}^{2}$ = 0.11).

### Gray Matter Volume

**Figure 3:**
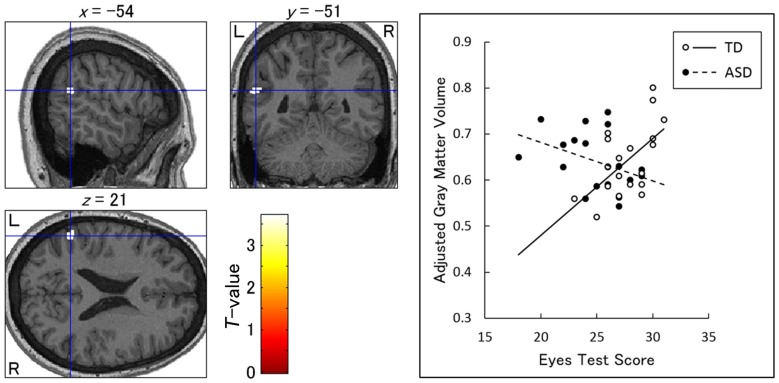
Brain regions showing a significant interaction between group and Eyes Test score in the association with gray matter volume. Left: statistical parametric maps showing the interaction in the left temporoparietal junction (posterior middle temporal gyrus). The height threshold was set at an uncorrected *p* < 0.001. The areas are overlaid on the normalized T1-weighted anatomical image of a study participant. Blue crosses indicate the locations of the peak voxels. The red–yellow color scale represents the *T*-value. L, left hemisphere; R, right hemisphere. Right: scatterplots of the adjusted gray matter volume as a function of Eyes Test scores at the peak voxels for the typically developing (TD) and autism spectrum disorder (ASD) groups. Effects of no interest (age, sex and full-scale intelligence quotient) were cavariated out.

Our ROI analyses revealed a significant group × Eyes Test score interaction (TD Eyes Test score vs. ASD Eyes Test score) in the left TPJ (posterior middle temporal gyrus; peak: *x*-54, *y*-51, *z*21; BA22, *T*_(31)_ = 3.35; Figure 3), indicating that the association between the Eyes Test score and gray matter volume in this region differed between groups such that the association was weaker in the ASD than in the TD group. The simple effect contrasts revealed a significant positive association between the Eyes Test score and gray matter volume only in the TD group. When we explored the data with a more liberal height threshold (*p* < 0.05, uncorrected) for descriptive purposes, there was a cluster showing a negative association between the Eyes Test score and gray matter volume of this region in the ASD group. We found no significant effects of age, sex and full-scale IQ in the identified TPJ region (Supplementary Figure S2).

Our search for interactions between group and Eyes Test score revealed no significant clusters in other regions of the brain. Furthermore, we found no significant clusters indicating main effects of group (group differences in brain volume regardless of Eyes Test score) or Eyes Test score (positive or negative associations with brain volume consistent across groups).

## Discussion

Our behavioral results showed that Eyes Test scores were lower in the ASD group than in the TD group. This result is consistent with several previous studies (e.g., Baron-Cohen et al., 1997) and indicates that individuals with ASD are impaired in their ability to read the others’ minds by viewing their eyes.

More importantly, our VBM results revealed an interaction between group and Eyes Test score in the left TPJ, indicating a weak association between the Eyes Test score and gray matter volume in the ASD group relative to the TD group. We found a positive relationship between the Eyes Test score and gray matter volume in the left TPJ of individuals with TD, but not in those with ASD; in fact, the association was slightly negative. These results are consistent with the findings of several functional MRI studies that the TPJ is active while TD individuals perform the Eyes Test (e.g., Platek et al., 2004; for a review, see Schurz et al., 2014) and that siblings of individuals with ASD showed less activation in the TPJ during the Eyes Test compared with the TD group (Holt et al., 2014). Our results appear to be inconsistent with those of a previous structural MRI study showing a positive relationship between social cognition and gray matter volume in the TPJ of individuals with ASD (David et al., 2014). However, methodological differences can account for this discrepancy. For example, in the David et al.’s (2014) study, the task was to rate interactions between non-human objects, and did not involve processing eyes or faces. Furthermore, the authors found no association between task performance and gray matter volume in the TPJ of TD individuals, suggesting that the region investigated was functionally different from that in our study. Our findings are consistent with several previous structural MRI studies showing weak and negative associations between the processing of facial stimuli and gray matter volume in the fusiform gyrus (Dziobek et al., 2010; Trontel et al., 2013) and amygdala (Dziobek et al., 2006), in individuals with ASD. The atypical, paradoxical association between social functioning and gray matter volume in individuals with ASD may be related to their compensatory cognitive (e.g., more intellectual) or biological (e.g., using different brain regions) strategies for social interaction. Taken together, our findings suggest that the failure to make appropriate structural neural representations in the TPJ may underlie the impaired ability of individuals with ASD to read the mind in the eyes.

Our results showing an interaction between group and Eyes Test score in the structure of TPJ are similar to the findings of previous structural MRI studies showing that adults with ASD had a structural abnormality in the TPJ (Hadjikhani et al., 2006; Craig et al., 2007; Scheel et al., 2011; Ecker et al., 2012; Mueller et al., 2013; David et al., 2014). However, note that our results did not show a main effect of group (i.e., group differences in gray matter volume regardless of Eyes Test score) in the TPJ, which is consistent with several structural MRI studies in adults with ASD (Abell et al., 1999; McAlonan et al., 2002; Schmitz et al., 2006, 2008; Wilson et al., 2009; Dziobek et al., 2010; Toal et al., 2010; Ecker et al., 2013; Lai et al., 2013; Bernhardt et al., 2014; Riedel et al., 2014; Balardin et al., 2015; Gebauer et al., 2015; Itahashi et al., 2015; Libero et al., 2015; for a review see Yang et al., 2016). Our results suggest that individuals with ASD may have atypical brain–behavior associations that cannot be detected using structural MRI data alone.

Our results have theoretical implications. First, the results add empirical support for the cognitive theory that impaired abilities to process eyes and to read others’ minds are core symptoms of ASD (Baron-Cohen, 1995). Our behavioral results confirm these deficits, and our MRI results revealed their structural neural underpinning. Next, the MRI results also provide support for the neuroscientific theory of an impaired social brain network in ASD (Emery and Perrett, 2000; Johnson et al., 2005; Pelphrey and Carter, 2008; Sato et al., 2012). Although the details differ across studies, the theory posits that abnormal structures and/or functions in the network of specific brain regions involved in processing social signals, including the TPJ, underlie the social malfunctioning in individuals with ASD. Our results confirm that the TPJ is a core social brain region impaired in individuals with ASD.

Several limitations of this study should be acknowledged. First, we used the Asian version of the Eyes Test. Although this version has the advantage of increased behavioral and neural sensitivity for Japanese participants (Adams et al., 2010), it has not been investigated extensively and lacks sufficient data regarding reliability and validity. Thus, further psychometric studies are necessary to validate the Asian version of the Eyes Test.

Second, our sample was small, and hence the results should be interpreted cautiously. Although the TPJ was the only brain region in which we found a significant interaction between group and Eyes Test score, null findings in the other ROIs, or in other brain regions, may be attributable to the lack of statistical power. In fact, we found an association between the Eyes Test score and gray matter volume in other brain regions, including the dmPFC and precuneus in a subsequent investigation of more TD participants (Sato et al., 2016). Future studies with larger samples of individuals with ASD may reveal the structural neural network underlying the impaired ability to read the mind in the eyes and provide a better understanding of the brain regions involved in compensatory processing as individuals with ASD attempt to make inferences about others’ mental states.

Third, the ASD group included only individuals with high-functioning ASD. However, our findings and those of several previous studies in TD individuals showed that Eyes Test performance was correlated with IQ (e.g., Peterson and Miller, 2012; for a review see Baker et al., 2014). Such data suggest the possibility that performance on the Eyes Test may be more severely impaired and its structural neural correlates more widespread in individuals with low- compared with high-functioning ASD. Further research is needed to determine whether the results can be extended to individuals with lower-functioning ASD.

Finally, the specific cognitive functions related to the TPJ remain unclear. Debate regarding this issue persists in functional neuroimaging studies of TD individuals (see Van Overwalle and Baetens, 2009). For example, it has been proposed that the TPJ might be involved in gathering cues to infer mental states (Gallagher and Frith, 2003) or inferring transient mental states (Van Overwalle, 2009). Furthermore, questions over which core cognitive functions can be assessed by the Eyes Test (e.g., mind reading vs. emotion recognition; Oakley et al., 2016) have sparked debate in the psychological literature. Future research investigating the association between more specific cognitive functions related to reading the mind in the eyes and the TPJ structure would deepen our understanding of social impairment in ASD.

In conclusion, our VBM analysis showed an interaction between group and Eyes Test score in the left TPJ. We found a positive relationship between the Eyes Test score and TPJ gray matter volume in individuals with TD, but not in those with ASD. This finding suggests that failure to develop appropriate structural neural representations in the TPJ may underlie the impaired ability of individuals with ASD to read the mind in the eyes.

## Author Contributions

WS, SU, TK and MT designed the research; WS, SU, TK, SY, RS, YK, MS and MT obtained the data; WS, TK, SY and MT analyzed the data; and all authors wrote the manuscript. All authors read and approved the final manuscript.

## Conflict of Interest Statement

The authors declare that the research was conducted in the absence of any commercial or financial relationships that could be construed as a potential conflict of interest.

## Footnotes

^1^http://www.fil.ion.ucl.ac.uk/spm

^2^http://dbm.neuro.uni-jena.de

^3^http://www.brain-development.org

^4^http://imaging.mrc-cbu.cam.ac.uk/imaging/BrodmannAreas

^5^http://www.mccauslandcenter.sc.edu/mricro/mricron/

## Supplementary Material

FIGURE S1 Schematic illustrations of the design matrix (lower matrix image) and contrast (upper bar graph) in the general linear model analysis. The design matrix contained group (typically developing [TD] and autism spectrum disorder [ASD]) and group-interacted Eyes Test score as the effect of interest factors, while age, sex and full-scale intelligence quotient (IQ) were the covariates of no interest. Our prediction was related to the TD Eyes Test score vs. ASD Eyes Test score contrast.

FIGURE S2 Scatterplots of adjusted gray matter volume showing the relationships between effects of no interest (age, sex and full-scale intelligence quotient [IQ]) and Eyes Test score for the typically developing (TD) and autism spectrum disorder (ASD) groups at the peak voxels for the interaction between group and Eyes Test score. The effects of group and Eyes Test score, and the effects of no interest were covariated out.
