# Supplementary figures and images for: Structural Correlates of Reading the Mind in the Eyes in Autism Spectrum Disorder
Source: Front Hum Neurosci. 2017 Jul 12;11:361. doi: 10.3389/fnhum.2017.00361 (PMC5506186; doi:10.3389/fnhum.2017.00361)

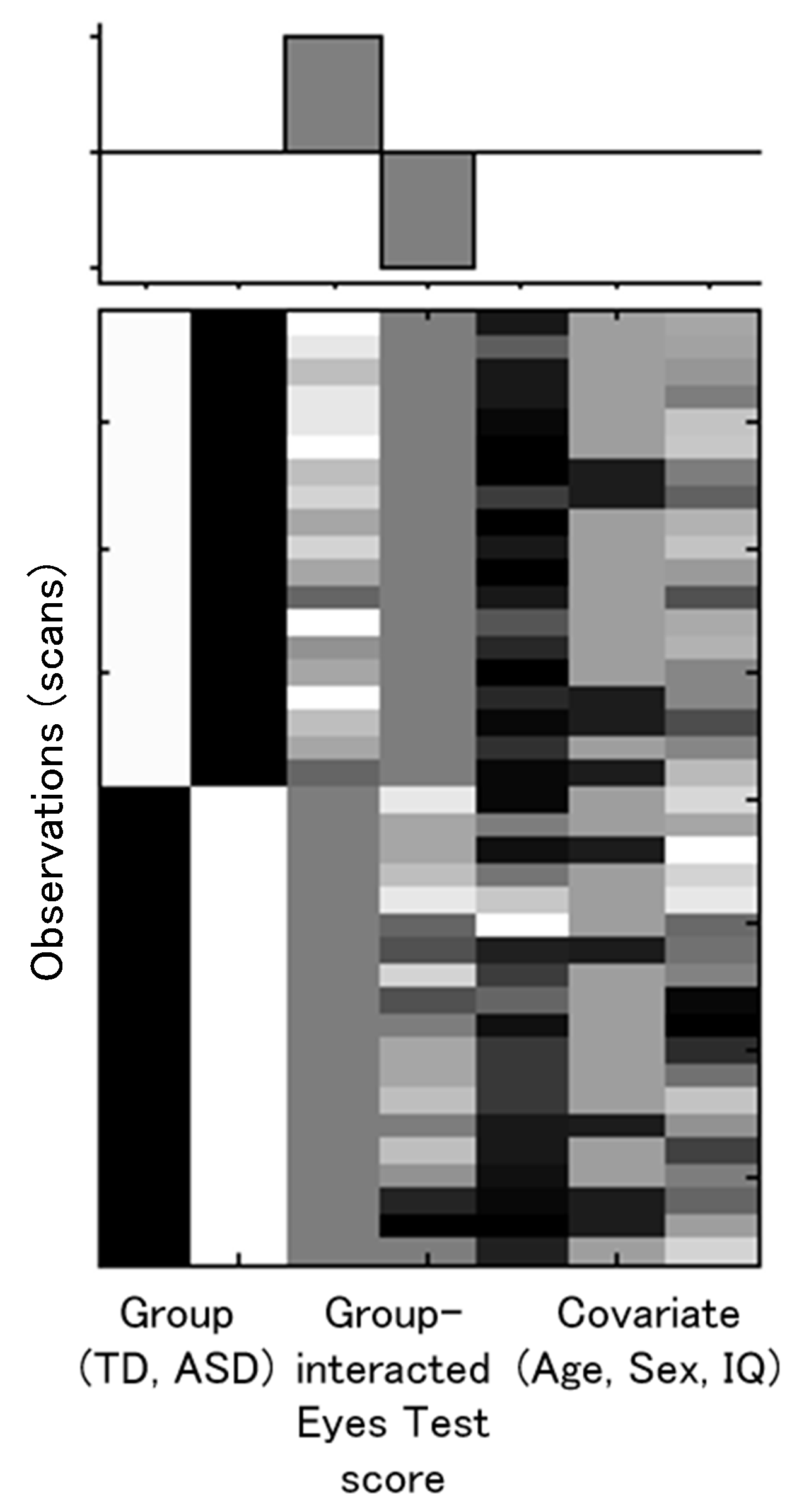

Supplement: FIGURE S1 — Schematic illustrations of the design matrix (lower matrix image) and contrast (upper bar graph) in the general linear model analysis. The design matrix contained group (typically developing [TD] and autism spectrum disorder [ASD]) and group-interacted Eyes Test score as the effect of interest factors, while age, sex and full-scale intelligence quotient (IQ) were the covariates of no interest. Our prediction was related to the TD Eyes Test score vs. ASD Eyes Test score contrast. [file Image_1.tif]

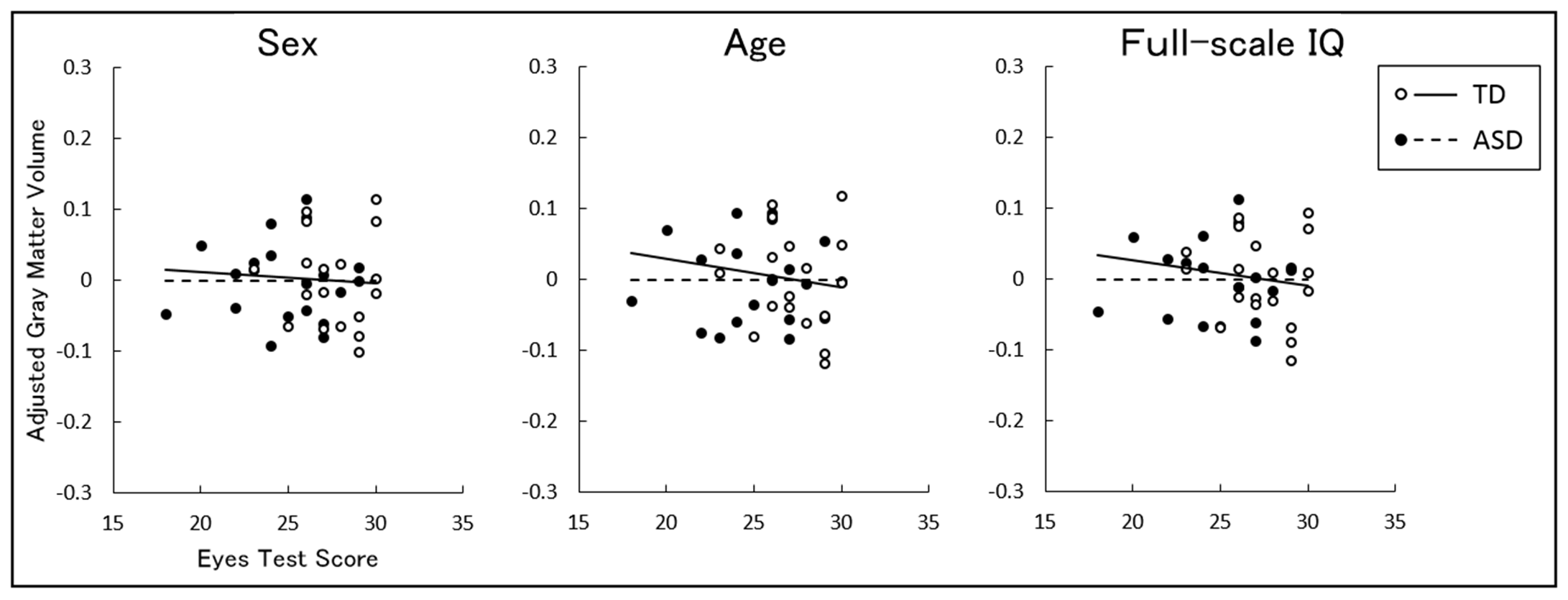

Supplement: FIGURE S2 — Scatterplots of adjusted gray matter volume showing the relationships between effects of no interest (age, sex and full-scale intelligence quotient [IQ]) and Eyes Test score for the typically developing (TD) and autism spectrum disorder (ASD) groups at the peak voxels for the interaction between group and Eyes Test score. The effects of group and Eyes Test score, and the effects of no interest were covariated out. [file Image_2.tif]
